# Evaluating the Effects of Different Cognitive Tasks on Autonomic Nervous System Responses: Implementation of a High‐Precision, Low‐Cost Complementary Method

**DOI:** 10.1002/brb3.70089

**Published:** 2024-10-08

**Authors:** Nazli Karimi Ahmadi, Sezgi Firat Ozgur, Erhan Kiziltan

**Affiliations:** ^1^ Department of Physiology, Faculty of Medicine Hacettepe University Ankara Turkey; ^2^ Department of Biophysics, Faculty of Medicine Baskent University Ankara Turkey

**Keywords:** autonomic nervous system (ANS), cognitive workload, heart rate variability (HRV), skin conductance response (SCR)

## Abstract

**Introduction:**

We developed a low‐cost, user‐friendly complementary research tool to evaluate autonomic nervous system (ANS) activity at varying levels of cognitive workload. This was achieved using visual stimuli as cognitive tasks, administered through a specially designed computer‐based test battery.

**Methods:**

To assess sympathetic stress responses, skin conductance response (SCR) was measured, and electrocardiograms (ECG) were recorded to evaluate heart rate variability (HRV), an indicator of cardiac vagal tone. Twenty‐five healthy adults participated in the study. SCR and ECG recordings were made during both tonic and phasic phases using a computer‐based system designed for visual stimuli. Participants performed a button‐pressing task upon seeing the target stimulus, and the relationship between reaction time (RT) and cognitive load was evaluated.

**Results:**

Analysis of the data showed higher skin conductance levels (SCLs) during tasks compared to baseline, indicating successful elicitation of sympathetic responses. RTs differed significantly between simple and cognitive tasks, increasing with mental load. Additionally, significant changes in vagally mediated HRV parameters during tasks compared to baseline highlighted the impact of cognitive load on the parasympathetic branch of the ANS, thereby influencing the brain–heart connection.

**Conclusion:**

Our findings indicate that the developed research tool can successfully induce cognitive load, significantly affecting SCL, RTs, and HRV. This validates the tool's effectiveness in evaluating ANS responses to cognitive tasks.

## Graphical abstract

We developed a low‐cost, user‐friendly tool to evaluate autonomic nervous system (ANS) responses under varying cognitive workloads. Our study showed that this tool effectively induced cognitive load, significantly impacting skin conductance levels (SCL), reaction times (RTs), and heart rate variability (HRV). The findings validate the tool's effectiveness in assessing ANS responses, highlighting its potential for broader research applications.

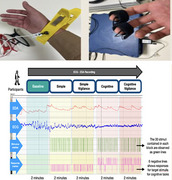

## 1 Introduction

The autonomic nervous system (ANS), composed of the sympathetic and parasympathetic systems, regulates bodily functions, balancing activity to facilitate survival responses and post‐threat recovery (McCorry 2007). Stress triggers sympathetic and parasympathetic responses, leading to changes in biomarkers and biosignals. On the basis of the literature review, heart rate variability (HRV) analysis and skin conductance response (SCR) are widely used as physiological indicators of stress (Battaglia et al. 2024; Jiménez‐Mijangos et al. 2023). According to the neurovisceral integration model, the ANS interacts dynamically with the central nervous system (CNS) to regulate heart function during cognitive load and fear (McEwen and Morrison 2013; Thayer and Lane 2009). When mental demands rise, the prefrontal cortex exerts top‐down control over the ANS, influencing heart rate via the vagus nerve (Di Gregorio et al. 2024). Simultaneously, the amygdala, critical in processing emotional stimuli, also impacts autonomic responses (Battaglia and Thayer 2022). This demonstrates how cognitive and emotional processes directly affect autonomic regulation, with HRV and SCR serving as key markers of cognitive load (Battaglia et al. 2024; Jiménez‐Mijangos et al. 2023). Coordinated CNS–ANS interactions are essential for survival, as social and emotional factors can significantly impact physiological responses during performance monitoring (Battaglia et al. 2024; Di Gregorio et al. 2024). SCR, also known as electrodermal activity (EDA), is a peripheral indicator of the sympathetic nervous system (SNS) activity (Dolu, Acer, and Kara 2014; Visnovcova et al. 2016). Skin conductance assessment measures alterations in electrical conductivity attributed to changes in sweat production (Boucsein et al. 2012). It serves as a physiological indicator for wakefulness and attention (Martínez Vásquez, Posada‐Quintero, and Rivera Pinzón 2023). Skin conductance measurements comprise two components: tonic (resting) and phasic (reactivity). Tonic skin conductance measures resting levels within seconds, whereas phasic responses involve brief oscillations triggered by stimuli such as fear or increased cognitive load, lasting a few seconds (Battaglia et al. 2024; Storm 2008). Particularly during mental tasks that elevate cognitive load, which is the mental effort used by working memory at any given time (Sazuka et al. 2024), combining skin conductance measurements with HRV metrics is recommended to comprehensively assess ANS responses (Nepal, Manandhar, and Jha 2019). HRV, a cardiac vagal tone indicator, is the fluctuation in the time intervals between successive heartbeats, known as inter‐beat intervals (IBIs) (Wettstein, Kühne, and Tschacher 2020). A healthy heart displays dynamic oscillations, allowing the cardiovascular system to promptly adapt to physical and psychological challenges. Higher HRV, indicating greater cardiac vagal tone and a flexible ANS, enhances cognitive performance (Zeng et al. 2023). HRV parameters can be derived through time–domain, frequency–domain, and nonlinear analyses (Chan et al. 2015). Mostly used time–domain HRV parameters are the root mean square of successive differences between normal heartbeats (rMSSD), the standard deviation of the successive IBIs for normal sinus beats (SDNN), and the percentages of successive IBIs for normal sinus beats differing by more than 10 ms (PNN10), 30 ms (PNN30), and 50 ms (PNN50) (Malik and Camm 1993). Among these parameters, rMSSD measures the activity of the parasympathetic nervous system (PNS) (Shaffer and Ginsberg 2017) and is assessed as vagal tone activity (Zeng et al. 2023). Understanding the coordination between SNS and PNS is crucial for enhancing the ability to organize resources effectively to meet various demands. Stenfors et al. discovered that higher rMSSD and higher SDNN were linked to better performance in cognitive tests, including working memory (Stenfors et al. 2016). However, this association weakened when age was considered a covariate (Zeng et al. 2023). Working memory, essential for learning and reasoning, influences cognitive function and performance (Yin et al. 2020; Yoo and Collins 2022). Previously, it was found that young individuals with higher resting HRV performed better in working memory tasks (Landolt et al. 2017). Chronic stress‐induced autonomic dysregulation can impair both decision‐making speed and reaction times (RTs). Mental tasks that demand attention can trigger stress, which, in turn, affects ANS responses and consequently alters RTs (Landolt et al. 2017). Alternating attention allows individuals to shift focus between tasks with varying cognitive demands (Kim et al. 2020), whereas sustained and selective attention enable consistent focus during continuous activities and amidst distractions (Moscatelli et al. 2024). The prefrontal cortex plays a key role in these processes, as neurophysiological studies in nonhuman primates have shown its involvement in functions like working memory, decision‐making, and action planning, highlighting its importance in managing complex cognitive tasks (Moscatelli, Monda, et al. 2023; Moscatelli, Toto, et al. 2023). Once responses to various levels of cognitive tasks are recorded along with electrophysiological signals such as SCR and electrocardiogram (ECG), ANS activity can be analyzed in relation to these tasks. To study stress levels and attention deficits in various conditions and diseases, it is crucial to use simpler, more affordable methods, especially when resources are limited (Nepal et al. 2018). Our study aimed to develop a reliable, cost‐effective tool to investigate the relationship between ANS activities and cognitive load. Using a custom‐built, computer‐based test battery, we evaluated the impact of different cognitive load levels as measured through ANS function. Specifically, we utilized visual stimuli as cognitive tasks, synchronizing the recording of EDA and ECG to assess HRV parameters and SCLs. This methodology enabled us to investigate how varying cognitive load levels influence SCL, RTs, and HRV indicators, as well as how internal time estimation affects stress levels and RTs during these tasks.

## 2 Materials and Methods

### 2.1 Participants

The study included healthy adults aged 20–40 who met specific inclusion criteria: no color vision deficiency, sufficient visual acuity to perceive shapes on a computer screen, the absence of neurological disorders, a minimum of 8 h of sleep the previous night, and minimum of 2 h since their last meal. The study exclusion criteria encompassed the presence of visual impairments, neurological disorders, fasting state, alcohol or substance addiction, and any cardiovascular disease. The size of the research group, participant characteristics, and study protocol were determined on the basis of similar studies (Setz et al. 2010). Twenty‐five right‐handed participants, with a mean age of 30.2 years (±10.42), were included in the study. The group consisted of 10 men and 15 women. Hand preference was determined using the Turkish version of the “Edinburgh Oldfield Inventory.” The results were analyzed according to the Geschwind score (Tan 1988). Color blindness was tested using the Ishihara color vision test (Pache et al. 2003). All measurements were performed in the same period of the day. The participants were instructed to press the predefined button located directly in front of them on the table with their dominant hand when they see the visual stimulus on the computer screen.

### 2.2 Experimental Setup

In order for evaluating, the effects of cognitive load on ANS activity, a cognitive performance test (CPT) battery has been integrated into the electrophysiological signal data acquisition system. This integration offers a holistic understanding of the relationship between cognitive load and ANS activity. Cognitive tasks used in the CPT that was already developed in our laboratory (Aydin et al. 2016; Karimi et al. 2023) consist of various visual stimuli.

### 2.3 Recording the Electrophysiological Signals

ECG and SCR signals were recorded using the Biopac MP36 Data Acquisition and Analysis System (BIOPAC Systems, Inc. CA, USA). The system provides valid, reliable, and safe electrophysiological data and allows for simultaneous analog signal recording up to four channels. ECG and SCR signals were recorded through the two channels of the system and saved for later analysis. For ECG recording, three electrodes are placed on the non‐dominant hand (left wrist), right ankle, and left ankle (Lead III configuration). SCR is recorded through electrodes attached to the fingertips of non‐dominant hand. ECG and SCR data are monitored on a computer screen that is visible only to the operator.

### 2.4 Experimental Procedures

**FIGURE 1:**
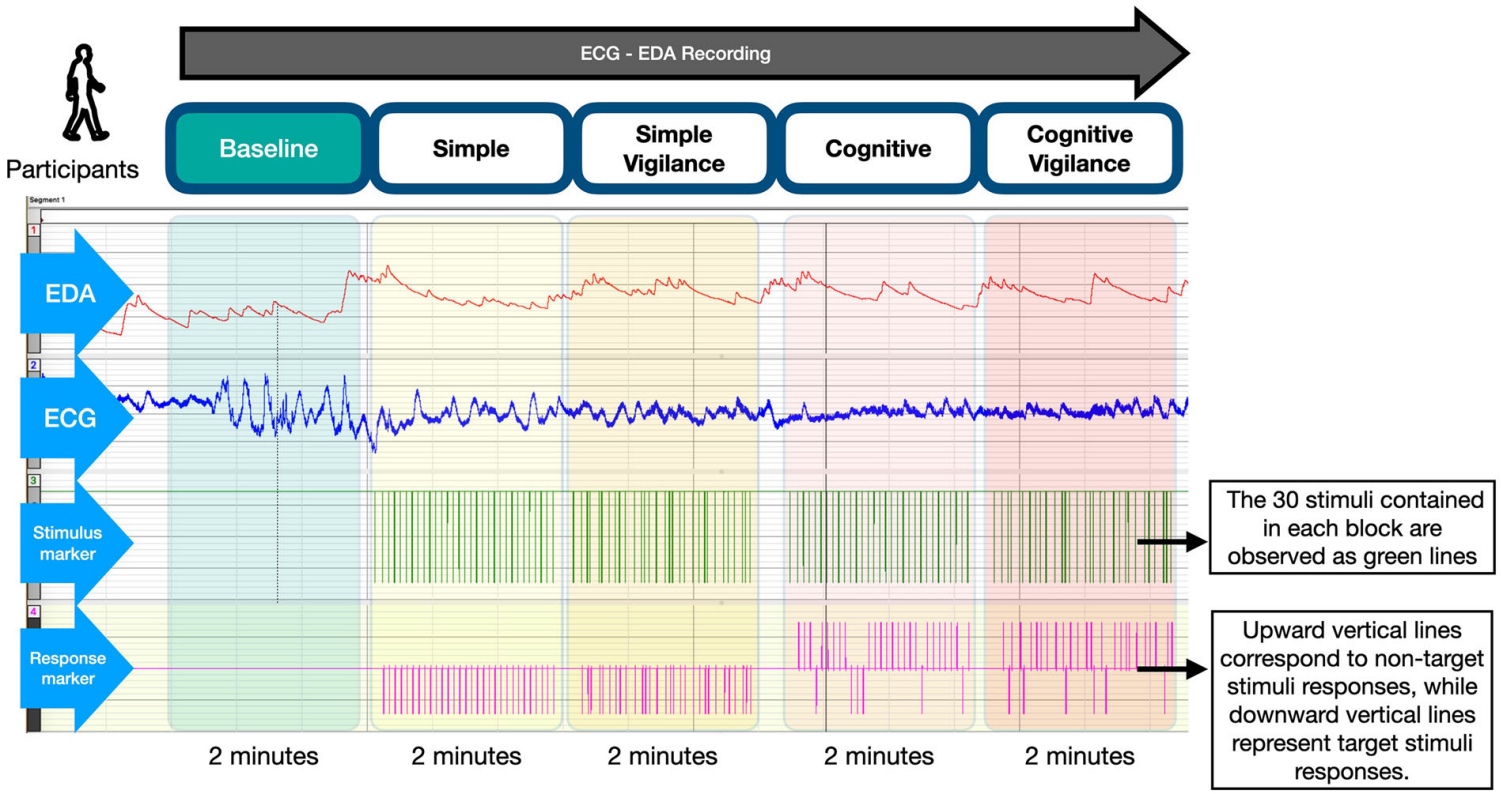
The Biopac system window displays five columns corresponding to the five blocks, and four rows representing EDA, ECG, stimulus, and response markers. first two rows of recording interface show EDA and ECG records. Row 3 shows each stimulus presented to the participants. Row 4 shows participants response to each stimulus via button. Upward vertical lines correspond to non‐target stimulus responses, whereas downward vertical lines represent target stimulus responses. On the recording screen, the baseline (resting state) and four task blocks are easily noticeable. ECG, electrocardiogram; EDA, electrodermal activity.

Participants were comfortably seated in the recording room and allowed to rest for 10 min before the test. They were informed about the experiment and instructed to remain still, avoid deep breaths, and maintain a relaxed posture throughout the test. They were instructed to remain still, avoid deep breaths, and maintain a relaxed posture throughout the test. The experimental procedure consisted of five steps, as detailed and outlined in Figure 1.

First, the skin conductance and ECG subunits were calibrated, followed by a 2‐min baseline recording in a resting state without stimulus. Subsequently, participants completed four tasks, each lasting 2 min and progressively increasing in cognitive load. Visual stimuli were used in each task, as previously described and summarized in Table 1 (Karimi et al. 2023). For this study, the number of stimulus and task durations were adjusted to refine the experimental design and optimize data collection and analysis. The tasks were categorized into two main types: simple and cognitive, each incorporating a vigilance component, resulting in four subgroups: simple, simple vigilance (SV), cognitive, and cognitive vigilance (CV). The vigilance tasks required heightened attention due to the randomly varying stimulus intervals.

**TABLE 1:** Visual tasks design description.

Tasks	Visual Stimulus	Stimulus Exposure Time (s)	Number of Stimulus	Inter Stimulus Interval (s)	Descriptions
Simple (S)	Simple RED	0.5	30	2 Fixed	Participant was asked to press on Button‐1 as quick as possible upon the onset of visual stimulus with index finger of the dominant hand
Simple vigilance (SV)	Simple RED	0.5	30	2–5 Random	Participant was asked to press on Button‐1 as quick as possible upon the onset of visual stimulus with the index finger of dominant hand
Cognitive (C)	Cognitive 5 color (red, black, green, yellow, blue)	0.5	24 Non‐target 6 Target	2 Fixed	Participant was asked to press on Button‐1 as quick as possible if the stimulus is red colored (target), otherwise Button‐2 with the index finger of dominant hand
Cognitive vigilance (CV)	Cognitive 5 color (red, black, green, yellow, blue)	0.5	24 Non‐target 6 Target	2–5 Random	Participant was asked to press on Button‐1 as quick as possible if the stimulus is red colored (target), otherwise Button‐2 with the index finger of dominant hand

*Note*: Non‐target stimulus: black, green, yellow, and blue; target stimulus: red.

### 2.5 Visual Stimuli

In the simple test, participants were shown red visual stimuli at either fixed or random intervals and were instructed to press a predefined button as soon as the stimulus appeared. In the cognitive test, participants were required to detect the colored visual stimulus, correctly identify it, and respond by pressing the corresponding button. Red was defined as the target stimulus, whereas other colors were non‐target stimuli.

### 2.6 Button Box

A specially designed, battery‐operated button box with two buttons (Buttons‐1 and 2) was used in the study. Each button generated a square wave signal with opposite polarity (+1 or −1 V) when pressed, which was input into the fourth analog input channel of the data acquisition system.

### 2.7 Synchronization

Visual stimuli were displayed on a secondary screen positioned in front of the participants. During stimulus presentation, a digital trigger signal was generated and sent to a USB‐connected trigger interface, which transmitted it to the third analog input channel of the data acquisition system. These trigger signals provided precise timestamps, enabling synchronization of the stimuli with ongoing ECG and skin conductance recordings, accurately marking the stimulus onset in the electrophysiological data.

### 2.8 Data Analysis

Participants’ physiological data, including mean SCL, ECG, and RTs, were collected using the Biopac MP36 system. The data were then transferred to MATLAB software (R2018a) for further analysis of EDA and HRV parameters across each task block for all participants. For SCL analysis, significant changes in skin conductance were identified following the presentation of visual stimuli (Dolu, Acer, and Kara 2014). From the ECG data, various HRV parameters were derived, including heart rate (BPM), SDNN, RMSSD, PNN10, PNN30, and PNN50. Given that time–domain HRV metrics often follow skewed distributions, we used the median as a robust measure to minimize the influence of extreme values, while also considering the mean to assess any observed differences.

### 2.9 Statistical Analysis

To standardize the data for analysis, each calculated parameter was expressed as a percentage change relative to the baseline block (resting state) value. Tables were prepared to show how participants’ physiological data changed across the four task blocks. First, we conducted repeated measures analysis to evaluate the effect of our tasks on participants’ RTs, as well as their SCL and HRV parameters. Next, participants were divided into two groups on the basis of their basal vagal tone, as indicated by baseline rMSSD values (low vs. high rMSSD). This grouping was performed using both the mean and median rMSSD values. We then analyzed whether there were significant differences in SCL and RTs between the low and high rMSSD groups. When the assumptions were met, repeated measures ANOVA was employed, with one between‐subject factor (group based on rMSSD) and one within‐subject factor (task). Descriptive statistics were reported as mean ± standard deviation. If the assumptions were not met, the Friedman test was used for comparisons between dependent groups (tasks), and the median (interquartile range) was presented as descriptive statistics. Significant results were further evaluated using post hoc comparisons with Bonferroni adjustment. Normality was assessed using the Shapiro–Wilk test, the homogeneity of variances using Levene's test, and sphericity using Mauchly's sphericity test. The significance level was set at *α* = 0.05. All statistical analyses were performed using IBM SPSS Statistics for Windows, Version 23.0 (Released 2015. Armonk, NY: IBM Corp)

## 3 Results

### 3.1 EDA Parameters

#### 3.1.1 Effects of Different Tasks on SCR

Mean SCL values were calculated for each task from the EDA data. Percentage change of SCL values relative to the baseline recording was assessed to evaluate the task‐induced changes. These percentage changes of SCL values were then used in statistical comparisons. Friedman analysis was employed to determine the percentage change in SCL values, revealing significant differences during the tasks (*p* = 0.006) (Table 2). SCL values for every task were found to be higher than the baseline recording. The percentage changes in SCL values showed significant differences. However, the percentage changes of SCL values during tasks with random inter‐trial intervals (ITI) (SV and CV) were lower than the percentage changes of SCL values observed in tasks with fixed ITI (S and C). The analysis revealed that the task blocks had a significant effect on SCL values with a partial eta squared value of 0.192, reflecting a moderate effect size (Cohen 2013). This finding indicates that the tasks elicited measurable changes in SCR.

**TABLE 2:** Comparing the effects of different tasks on the percentage change in skin conductance level (SCL) values.

Tasks	Median of percent change of SCL and (interquartile range values)
Simple (S)	122% (167)
Simple vigilance (SV)	82% (145)
Cognitive (C)	137% (112)
Cognitive vigilance (CV)	75% (74)

*Note*: Values represent the median of percent changes relative to the baseline value. Positive values indicate an increase relative to the baseline as a percentage change.

#### 3.1.2 Effects of Different Tasks on RTs

The participants’ RTs to the tasks are detailed in Table 3. In cognitive tasks, RTs to target and non‐target stimuli are analyzed separately, resulting in six distinct RT values for each participant. The RTs significantly differed between simple tasks (S and SV) and cognitive tasks (C and CV). Despite the absence of a significant difference in cognitive tasks, participants’ RTs tended to be longer for target stimuli compared to non‐target stimuli. The analysis showed a significant effect of task blocks on RTs, with a partial eta squared value of 0.837. This large effect size suggests that the cognitive load of the tasks substantially influenced participants’ RTs.

**TABLE 3:** Comparing the effects of different tasks on participants’ reaction times (RTs) to stimuli.

Tasks	Medians of RTs (ms) and (interquartile range values)
Simple (S)	544 (75)
Simple vigilance (SV)	569 (61)
Cognitive target (Ct)	737 (110)
Cognitive non‐target (Cn)	709 (61)
Cognitive vigilance target (CVt)	754 (91)
Cognitive vigilance non‐ target (CVn)	735 (71)
*p* < 0.001.	

### 3.2 Effect of Different Tasks on ECG‐derived Parameters

#### 3.2.1 Effect of Different Tasks on BPM, SDNN, and PNN

The percentage change in values for all parameters was compared to the baseline to evaluate task‐induced alterations. PNN10, SDNN, and BPM percent changes did not differ significantly across tasks (Boucsein et al. 2012; Hansen, Johnsen, and Thayer 2009; Setz et al. 2010). However, there were notable differences in the percentage changes of PNN30 (*p* = 0.06) and PNN50 (*p* = 0.021) (Table 4).

**TABLE 4:** Comparing the effects of different tasks on the percentage changes in PNN30 and PNN50 values.

Tasks	PNN30 percentage change and (interquartile range values)	PNN50 percentage change and (interquartile range values)
Simple (S)	7% (91)	62% (218)
Simple vigilance (SV)	−7% (55)	−2% (86)
Cognitive (C)	18 % (126)	41% (341)
Cognitive vigilance (CV)	−3% (77)	−13% (179)
	*p* = 0.006	*p * = 0.021

*Note*: PNN30: Percentages of successive IBIs for normal sinus beats differing by more than 30 ms; PNN50: percentages of successive IBIs for normal sinus beats differing by more than 50 ms. Values represent the median of percent changes relative to the baseline value. Positive values indicate an increase relative to the baseline as a percentage change. Negative values indicate a decrease relative to the baseline as a percentage change.

Specifically, we observed statistical variances between SV and C tasks, as well as between C and CV tasks in PNN30. Moreover, differences were evident between SV and C tasks in PNN50.

#### 3.2.2 Effects of Different Tasks on rMSSD

The percentage change of rMSSD values compared to the baseline was then analyzed to assess task‐induced changes. Friedman analysis indicated significant differences in the percentage change of rMSSD values during the tasks (*p* = 0.042) (Table 5). In pairwise comparisons, it was found that the main difference lies between S and CV tasks.

**TABLE 5:** Comparing the effects of different tasks on the percentage changes in RMSSD values.

Tasks	rMSSD percentage change and (interquartile range values)
Simple (S)	12 % (36)
Simple vigilance (SV)	−6 % (25)
Cognitive (C)	10 % (40)
Cognitive vigilance (CV)	−4 % (45)
*p *= 0.042	

*Note*: rMSSD: root mean square of successive differences between normal heartbeats. Values represent the median of percent changes relative to the baseline value. Positive values indicate an increase relative to the baseline as a percentage change. Negative values indicate a decrease relative to the baseline as a percentage change.

#### 3.2.3 Comparisons Between High and Low Groups Based on Baseline rMSSD Values

Comparisons were conducted between high and low groups on the basis of baseline rMSSD values. This grouping was performed in two distinct manners, using both the mean and median rMSSD values. The mean baseline RMSSD value for participants was 30.39, whereas the median baseline rMSSD value was 24.

#### 3.2.4 Comparison of SCL Values and RTs between Low and High rMSSD Groups

**FIGURE 2:**
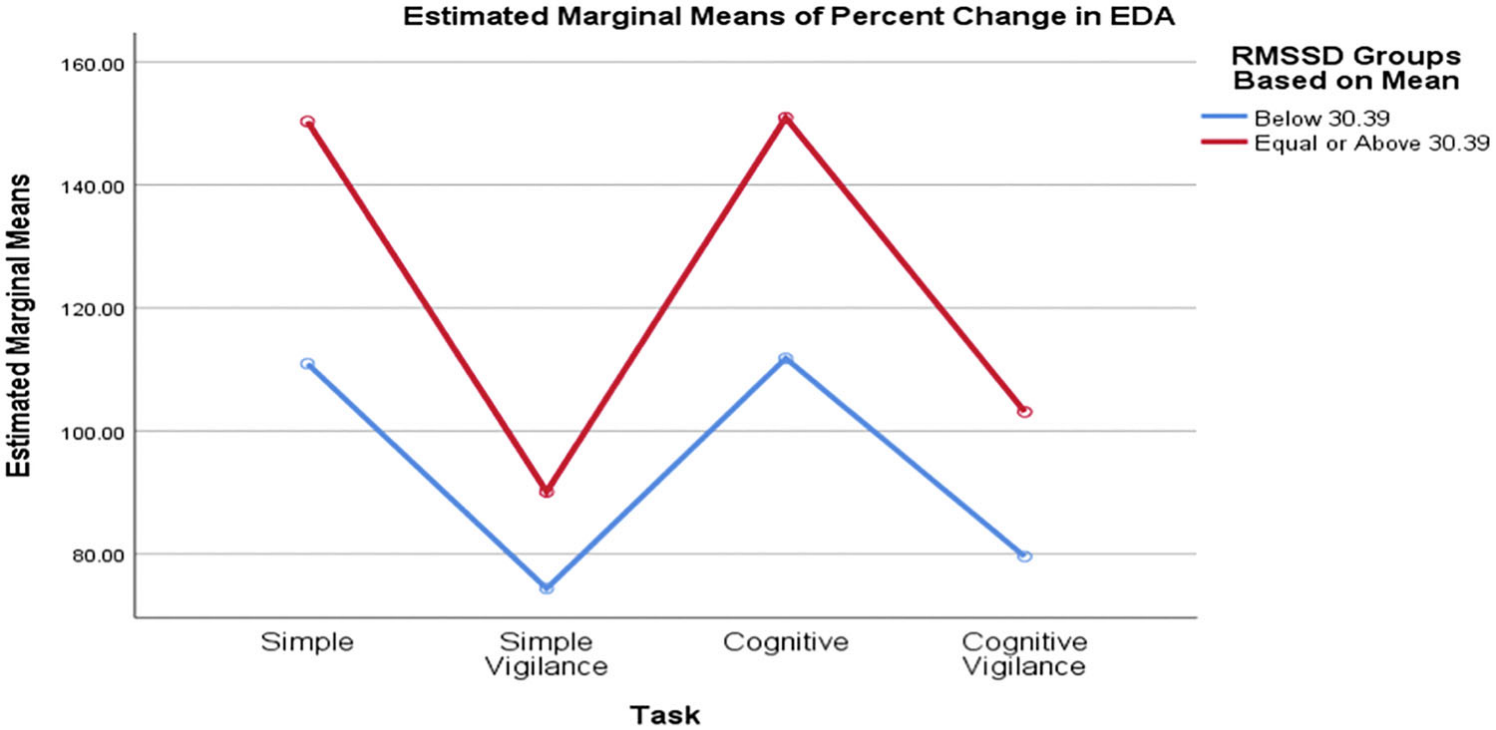
The percentage changes in participants’ SCL in response to different tasks remained consistent across both low and high rMSSD groups (based on mean), showing no significant differences. EDA, electrodermal activity; rMSSD, root mean square of successive difference; SCL, skin conductance level.

**FIGURE 3:**
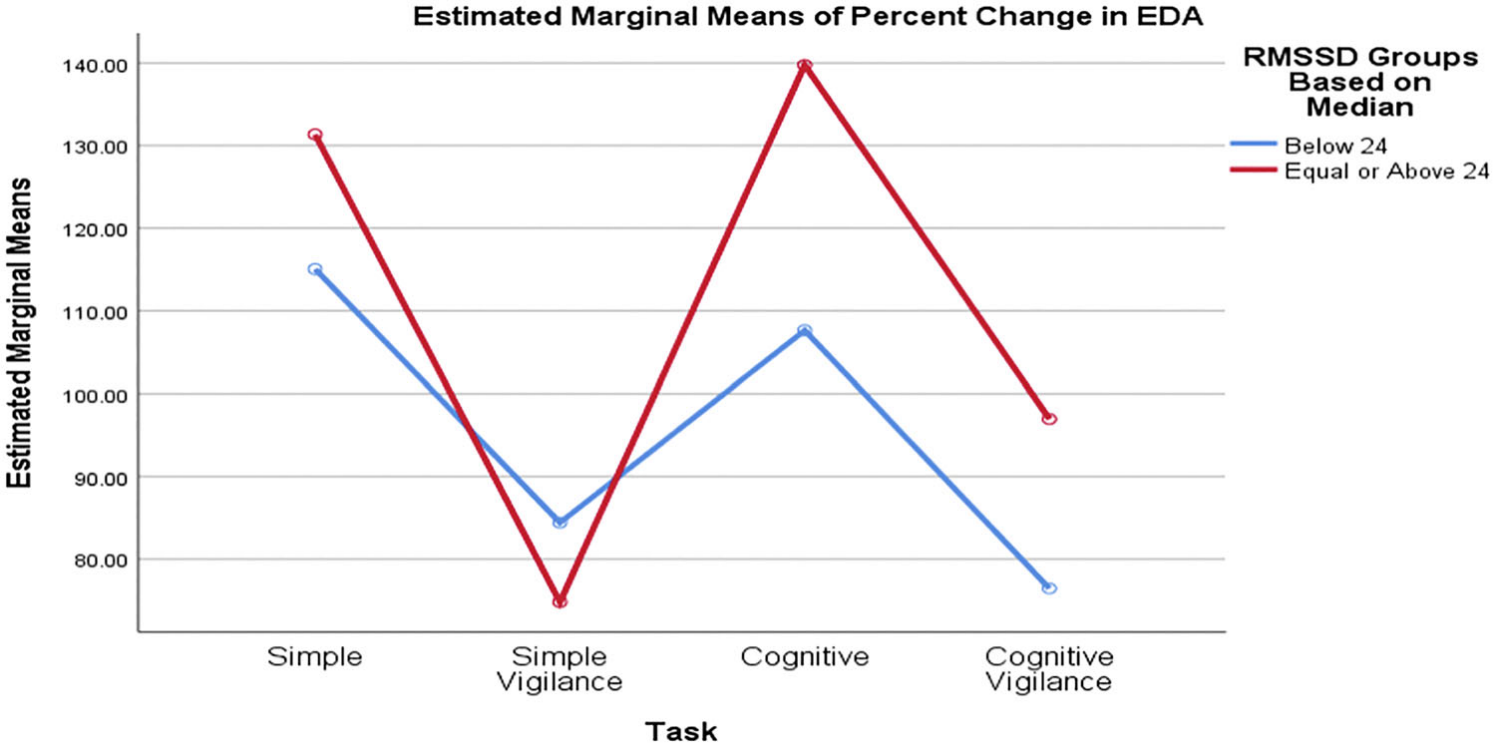
The percentage changes in participants’ SCL in response to different tasks remained consistent across both low and high rMSSD groups (based on median), showing no significant differences. EDA, electrodermal activity; rMSSD, root mean square of successive difference; SCL, skin conductance level.

Comparing groups formed based on the median baseline rMSSD value, the SCL percentage changes differed significantly across various tasks. Although the task significantly affected the percentage change in SCL (p_task = 0.002, interaction = 0.530, p_RMSSD_group = 0.635), the RMSSD groups had no statistically significant effect, nor did the interaction (Figure 2). Similarly, when comparing SCL values between low and high RMSSD groups based on the mean baseline RMSSD value, the task significantly affected the percentage change in SCL (p_task = 0.002, interaction = 0.837, p_RMSSD_group = 0.376), but the rMSSD groups and the interaction effect were not statistically significant (Figure 3).

#### 3.2.5 Comparison of RTs Values between Low and High Groups

**FIGURE 4:**
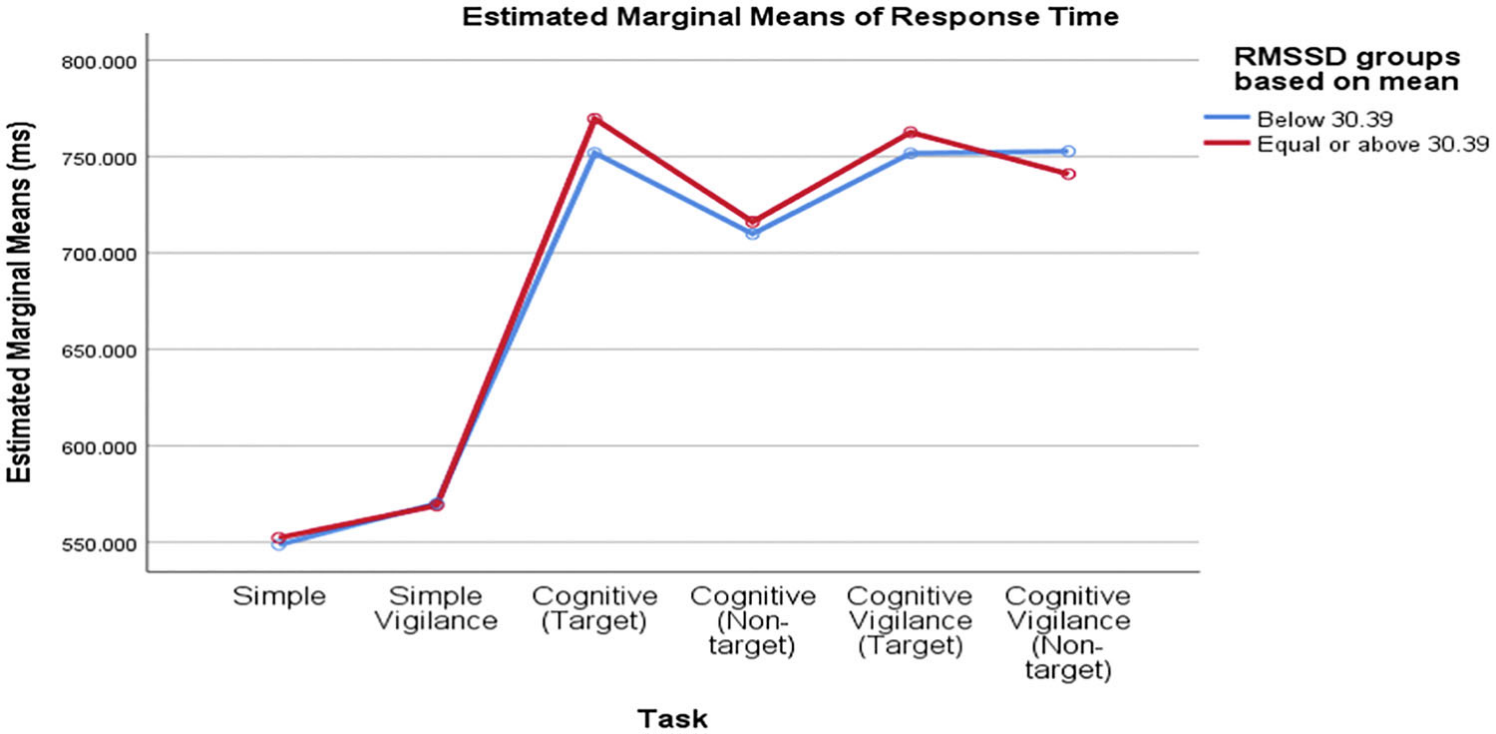
The changes in participants’ RTs in response to different tasks were consistent across both low and high rMSSD groups (based on mean), with no significant differences observed. rMSSD, root mean square of successive difference; RT, reaction time.

**FIGURE 5:**
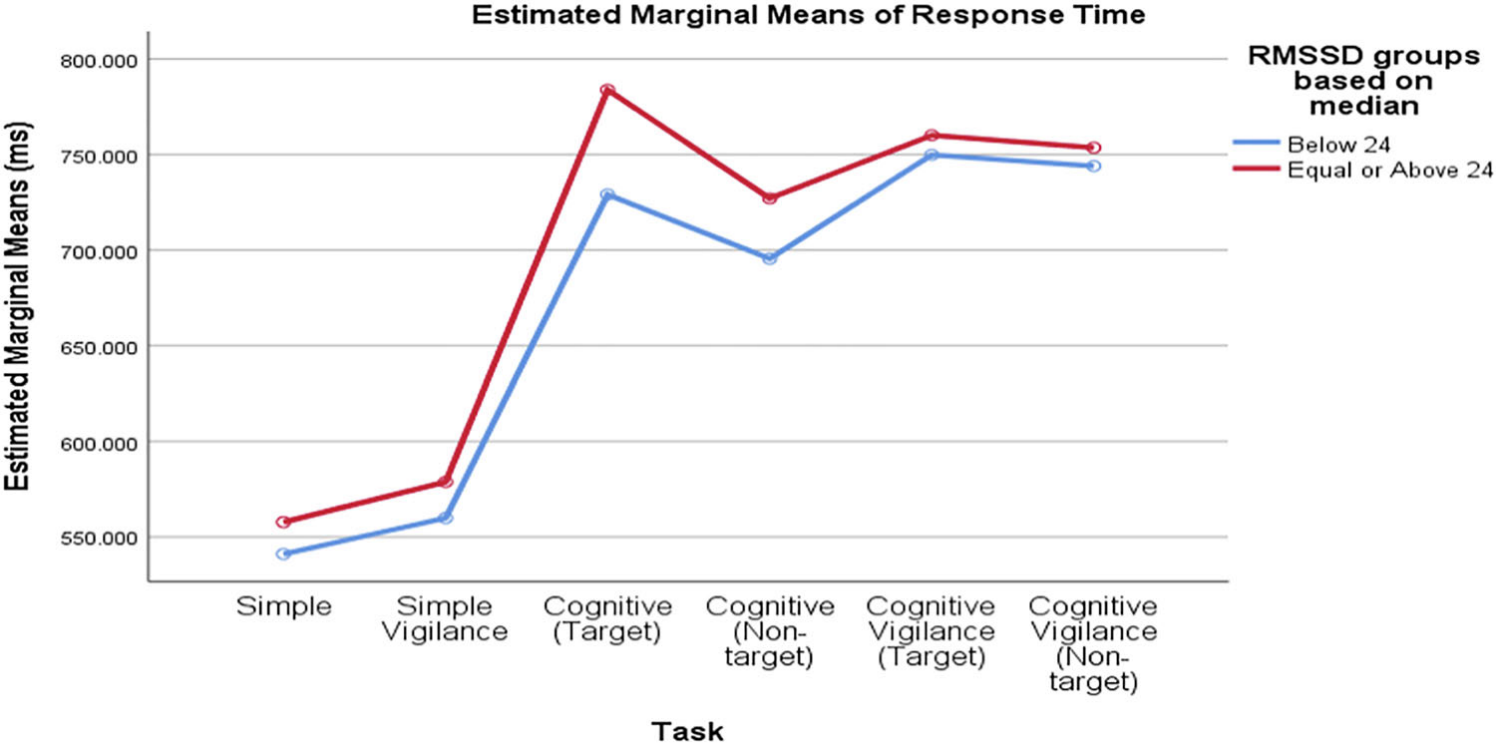
The variations in participants’ RTs across different tasks were consistent among both low and high rMSSD groups (based on median), with no significant differences detected. rMSSD, root mean square of successive difference; RT, reaction time.

Comparing groups based on the median baseline rMSSD value, RTs percentage changes differed significantly across tasks. The task significantly affected the percentage change in SCL (p_task < 0.001), but the rMSSD groups and the interaction did not (interaction = 0.356, p_rMSSD_group = 0.247; Figure 4). Similarly, when comparing RTs values between low and high RMSSD groups based on the mean baseline RMSSD value, the task significantly affected the percentage change in SCL (p_task < 0.001), but the RMSSD groups and the interaction were not significant (interaction = 0.814, p_rMSSD_group = 0.847; Figure 5).

## 4 Discussion

Our analysis showed that participants’ SCL increased during the tasks, indicating that these tasks effectively triggered SNS activation, resulting in heightened arousal and stress. This outcome is in consistent with the earlier studies. Nepal et al. demonstrated that skin conductance is a reliable indicator of sympathetic activation during cognitive stressors such as mental subtraction tasks. Similarly, Remier et al. observed elevated SCR during cognitive tasks, further supporting this relationship. Additionally, Kindermann et al. reported increased SCR during stress periods, and Scavone et al. found a strong correlation between SCL and emotional stress, reinforcing the role of SCR as a marker for sympathetic activity across different types of stressors (Kindermann and Werner 2014; Nepal et al. 2018; Reimer and Mehler 2011; Scavone, Kadziolka, and Miller 2020).

HRV is a well‐established marker of parasympathetic activity (Shaffer and Ginsberg 2017; Zeng et al. 2023). Previous studies have shown that individuals with anxiety disorders tend to have lower resting‐state HRV (Chalmers et al. 2014; Cheng et al. 2022). In our study, significant changes in vagally mediated HRV during tasks, compared to resting state, highlight the impact of cognitive load on the parasympathetic branch of the ANS. Although previous studies focused on tonic HRV, our research examined phasic HRV, which measures the change in HRV from baseline to task periods, offering deeper insight into how HRV fluctuations during cognitive tasks relate to performance.

The examination of RTs further supports the influence of cognitive load on physiological responses. We found significant differences in RTs between simple and cognitive tasks, with RTs increasing in parallel with the rise in mental load. This finding aligns with our previous study and other research, which suggests that more complex cognitive tasks require greater cognitive resources, leading to longer processing times and delayed responses (Fan et al. 2020; Karimi et al. 2023; Parrington, MacMahon, and Ball 2015).

The detailed aspects of our results include as follows:

SCL changes:

The percentage change in SCL from baseline recordings was found to be higher in vigilance tasks compared to their non‐vigilance counterparts. The vigilance tasks were designed to heighten participants’ alertness, leading to an increased stress response. However, as participants initially engaged in non‐vigilance tasks before transitioning to vigilance tasks, it was anticipated that sympathetic stress would diminish over subsequent task sessions. These findings align with Steptoe and Greer's 1980 study, which observed lower levels of autonomic arousal as training progressed (Steptoe and Greer 1980). Additionally, the internal timing processes used to estimate event occurrences in non‐vigilance tasks may create a greater cognitive load, impacting the sympathetic response more than simply awaiting random stimuli, as occurs in vigilance tasks (Dolu et al. 2004; Golob and Starr 2000). Moreover, to determine the cause of the observed decrease in SCL values during vigilance tasks, the order of the tasks can be changed to rule out the influence of learning effects.

RTs changes:

As anticipated, RTs increased with task difficulty. Although there was a trend toward longer RTs for target stimuli, this difference did not reach statistical significance. The lower frequency of target stimuli, which occurred at least four times less often than non‐target stimuli, likely contributed to slower RTs by reducing the activation of rapid decision‐making processes. This observation is consistent with findings by Lucci et al., who reported that a higher frequency of target stimuli is associated with faster RTs (Lucci et al. 2016). However, it is important to consider potential confounding variables that could influence these results. For instance, individual differences in baseline ANS activity may have affected the relationship between cognitive load and RTs. For example, in our study, the age of participants, which ranged from 20 to 40 years, could also influence ANS activity and, consequently, the response patterns under cognitive load. This suggests that future studies could benefit from categorizing participants by age to better understand these effects.

### 4.1 Vagally Mediated HRV Parameters Change

Previous studies have observed that increased vagally mediated cardiac control enhances the ability to quickly adapt to changing demands, thereby improving behavioral regulation and cognitive performance (Hansen, Johnsen, and Thayer 2003; Laborde, Furley, and Schempp 2015; Zeng et al. 2023).

As mentioned earlier, our study focused on phasic HRV, providing a clearer understanding of how HRV fluctuations during cognitive tasks impact performance. There were notable differences in the percentage changes of PNN30, PNN50, and rMSSD during tasks compared to the baseline values. When analyzing the data, we observed that non‐vigilance tasks led to an increase in HRV parameters compared to resting‐state values. In contrast, vigilance tasks showed a tendency for a decrease in HRV parameters relative to the resting state. Pairwise comparisons of rMSSD values revealed that the main difference was between the S and CV tasks, with rMSSD values decreasing as task difficulty increased. This aligns with the previous research showing that acute or chronic stress increases sympathetic dominance, leading to higher heart rate and lower HRV. Conversely, relaxation and recovery increase parasympathetic activity, resulting in lower heart rate and higher HRV (Kim et al. 2018; Pham et al. 2021).

### 4.2 Changes in SCL and RTs Based on High and Low Baseline rMSSD Values

Our study's analysis of participants grouped by baseline rMSSD values indicates that task‐induced changes in RTs and SCL are primarily attributable to cognitive load variations. However, research findings on this topic vary. In the research by Zeng et al., high vagally mediated resting‐state HRV was linked to better working memory and faster RTs (Zeng et al. 2023). Similarly, Hansen et al. found that participants with high HRV demonstrated better accuracy on cognitive tests, regardless of environmental conditions. However, the low HRV group exhibited faster RTs under threat‐of‐shock conditions (Hansen, Johnsen, and Thayer 2009). Conversely, some studies, consistent with our findings, found no significant difference between low and high resting HRV groups; for example, Williams et al. reported no significant correlation between performance on the working memory task and resting‐state vagal tone (Williams et al. 2019). The link between high vagally mediated resting‐state HRV, shorter RTs, and better cognitive performance remains debated. These controversies may arise from differences in task designs, types of stimuli, and tests used in research, as various stimuli can differently impact ANS activity. Although skin conductance and time–domain HRV parameters reflect sympathetic and parasympathetic activity, respectively, they assess different organs, and sympathetic outflow is not uniform (Bouvier and de Champlain 1985). Therefore, skin conductance does not necessarily indicate changes in sympathetic myocardial activity. Both measures are sensitive to task demands, but their relative sensitivity can vary depending on the type of task.

## 5 Conclusion

Our findings indicate that cognitive tasks increase sympathetic outflow, causing significant changes in SCL and RTs, and significantly affect the brain–heart connection, leading to notable changes in HRV. Our system's flexibility in modifying task parameters, such as type, duration, and stimulus intervals, makes it ideal for analyzing ANS responses under cognitive load. Future research studies could utilize this system to explore the impact of cognitive load on physiological responses, including changes in ANS function during aging, as previously mentioned. It is also well‐suited for investigating ANS activity across a range of conditions, including psychiatric disorders like attention deficit hyperactivity disorder (ADHD) and non‐psychiatric conditions such as cardiologic diseases, where ANS dysregulation is prevalent.

## Author Contributions

**Nazli Karimi Ahmadi**: conceptualization, investigation, methodology, validation, writing–review and editing, formal analysis, project administration, data curation, supervision, writing–original draft, visualization, software. **Sezgi Firat Ozgur**: conceptualization, methodology, writing–review and editing, software, formal analysis, validation. **Erhan Kiziltan**: conceptualization, investigation, methodology, writing–review and editing, software, formal analysis, validation.

## Ethics Statement

This study has received approval from the Hacettepe University Institutional Review Board and Ethics Committee under the project number GO 23/234 and decision number 2023/07‐49. The study conforms with The Code of Ethics of the World Medical Association (Declaration of Helsinki).

## Consent

Written informed consent was obtained from all participants prior to the initiatiion of the study.

## Conflicts of Interest

The authors declare no conflicts of interest.

### Peer Review

The peer review history for this article is available at https://publons.com/publon/10.1002/brb3.70089.

## Data Availability

The data that support the findings of this study are available from the corresponding author upon reasonable request.
