# Correction to Evaluating the Effects of Different Cognitive Tasks on Autonomic Nervous System Responses: Implementation of a High‐Precision, Low‐Cost Complementary Method

**DOI:** 10.1002/brb3.70203

**Published:** 2025-02-04

N. K Ahmadi, S. F. Ozgur, and E. Kiziltan, “Evaluating the Effects of Different Cognitive Tasks on Autonomic Nervous System Responses: Implementation of a High‐Precision, Low‐Cost Complementary Method.” Brain and Behavior 14, no. 10 (2024): e70089, https://doi.org/10.1002/brb3.70089

The first paragraph in Section 2.4 contained duplicate text. The corrected paragraph is as follows:

Participants were comfortably seated in the recording room and allowed to rest for 10 min before the test. They were informed about the experiment and instructed to remain still, avoid deep breaths, and maintain a relaxed posture throughout the test. The experimental procedure consisted of five steps, as detailed and outlined in Figure 1.

Section 3.1 erroneously contained reference citations to Boucsein et al. (2012); Hansen, Johnsen, and Thayer (2009); and Setz et al. (2010). The corrected text is as follows:

The percentage change in values for all parameters was compared to the baseline to evaluate task‐induced alterations. PNN10, SDNN, and BPM percent changes did not differ significantly across tasks. However, there were notable differences in the percentage changes of PNN30 (*p* = 0.06) and PNN50 (*p* = 0.021) (Table 4).

The online version of this article has been corrected accordingly.

We apologize for this error.

